# The impact of inotersen on Neuropathy Impairment Score in patients with hereditary transthyretin amyloidosis with polyneuropathy

**DOI:** 10.1186/s12883-023-03116-7

**Published:** 2023-03-17

**Authors:** Aaron Yarlas, Andrew Lovley, Duncan Brown, Montserrat Vera-Llonch, Sami Khella, Chafic Karam

**Affiliations:** 1 QualityMetric Incorporated, LLC, 1301 Atwood Avenue, Suite 216E, Johnston, RI 02919 USA; 2 Ionis Pharmaceuticals, Boston, MA USA; 3 University of Pennsylvania, Philadelphia, PA USA

**Keywords:** Neuropathy, Hereditary transthyretin amyloidosis, Neuropathic progression, Inotersen

## Abstract

**Background:**

Patients with hereditary transthyretin amyloidosis (ATTRv) frequently experience symptoms of polyneuropathy (PN) that worsen over time and impair daily functioning. Previous analyses supported efficacy of inotersen, an antisense oligonucleotide, to slow neuropathic progression in patients with ATTRv-PN, as indicated by larger mean changes, relative to placebo, in total score and several subscales of the Neuropathy Impairment Score (NIS), and for the subset of NIS items specific to lower limbs (NIS-LL) for the overall study sample. A key objective of the current study was to evaluate efficacy of inotersen for slowing neuropathic progression in NIS/NIS-LL within key clinical subgroups of patients with ATTRv-PN. Additionally, for this study, responder definition (RD) thresholds were estimated for NIS/NIS-LL total and subscale scores, for the purpose of evaluating clinically meaningful benefit of inotersen at the individual patient-level.

**Methods:**

Post hoc analyses used data from the NEURO-TTR phase 3 trial of inotersen in patients with ATTRv-PN (NCT01737398). Treatment differences in mean changes on NIS/NIS-LL total and subscale scores from baseline to week 65 were examined within patient subgroups defined by clinical characteristics. Anchor- and distribution-based approaches estimated RDs for NIS/NIS-LL scores, with responders defined as patients who did not experience clinically meaningful neuropathic progression. Responder analyses compared the proportion of patients classified as responders for each NIS/NIS-LL score between treatment arms.

**Results:**

Within each patient subgroup, mean increases in NIS/NIS-LL total and muscle weakness subscales were significantly smaller after 65 weeks of treatment with inotersen compared to placebo. Similar patterns were observed for some, but not all, subgroups on NIS/NIS-LL reflex subscale scores. Recommended RDs were 8.1 points for NIS total and 4.7 points for NIS-LL total. Patients receiving inotersen for 65 weeks were significantly less likely than those receiving placebo to exhibit clinically meaningful increases on NIS/NIS-LL total, muscle weakness, and sensation subscales.

**Conclusions:**

This study supports previous evidence for efficacy of inotersen in this patient population and provides interpretation guidelines for clinically meaningful changes in NIS/NIS-LL scores.

**Supplementary Information:**

The online version contains supplementary material available at 10.1186/s12883-023-03116-7.

## Background

Hereditary transthyretin amyloidosis (ATTRv) is a rare, systemic, progressive, and life-threatening disease caused by the misfolding of the transthyretin (TTR) protein and consequent formation of amyloid fibrils, which deposit in organs and tissues throughout the body and disrupt their ability to function [1, 2]. The accumulation of TTR amyloid in nervous tissue often leads to polyneuropathy (PN), manifesting as sensorimotor impairment and autonomic dysfunction that worsens rapidly over time without treatment [3]. Common symptoms such as numbness, fatigue, and weakness in the limbs can increasingly limit patients’ independence and ability to carry out daily activities, with substantial impact on their quality of life [4–6].

Current gene-silencing treatments for patients with ATTRv-PN aim to slow or halt further damage to organs and tissues, and worsening symptoms, by limiting the production of new TTR amyloid [7]. In the phase 3 NEURO-TTR trial [8], patients with ATTRv-PN receiving inotersen, an antisense oligonucleotide, exhibited slower progression of neuropathic symptoms, relative to patients receiving placebo, after 65 weeks of treatment, as measured by statistically significant treatment differences in change on the muscle weakness, sensation loss, and reflex subscales of the clinician-reported Neuropathy Impairment Score (NIS) and the NIS-Lower Limbs (NIS-LL), with the latter comprised of a subset of NIS items specific to the lower limbs [9]. However, while treatment differences were observed for the total sample, the benefit of inotersen on changes in NIS and NIS-LL scores within subgroups of patients with ATTRv-PN defined by key clinical characteristics has not yet been evaluated. ATTRv-PN is a highly heterogeneous disease, with differences in genetic mutation, organ involvement, stage of disease progression, and symptoms, contributing to a diversity of patient experiences. Evaluating the efficacy of treatment among patient subgroups defined by these clinical characteristics could show where treatment benefit is greatest, as well as help identify areas of unmet need.

Further, while the benefit of inotersen on NIS and NIS-LL was shown to be statistically significant at the group level, whether this benefit was clinically meaningful at the level of the individual patient has not yet been investigated. Responder definition (RD) thresholds, also referred to as the minimal clinically important difference (MCID), have been defined as the smallest difference in score that patients would consider a benefit and would warrant a change in their treatment [10]. While some researchers have proposed RD thresholds in NIS or NIS-LL scores, these values are based on a misinterpretation of the literature. For example, researchers have stated that the minimal clinically meaningful change is a 2-point increase (i.e., worsening) on the NIS-LL total score [11], which has a score range of 88 points, while others have stated that this 2-point threshold applies to the NIS plus 7 nerve tests (NIS + 7) [12], which has a score range of 240 points, or the modified NIS + 7 (mNIS+ 7) [8], which has a score range of 369 points, despite the fact that all three cite the same source for these values [13]. Further, the cited source actually refers to a 2-point change on the NIS total score, which has a range of 244 points, as indicating meaningful change, although no empirical evidence is provided to support this value, and this threshold was not estimated with respect to patients with ATTRv-PN [13]. A 2-point change represents a change of 0.5% on the mNIS+ 7, a change of 0.8% on the NIS or NIS + 7, and a change of 2.3% on the NIS-LL, which are all far lower than what is typically observed for the magnitude of an RD threshold. As such, there are currently no established, empirically supported RD thresholds that represent meaningful change in NIS or NIS-LL scores, limiting the degree to which a treatment benefit can be evaluated as providing a clinically meaningful benefit to patients with ATTRv-PN.

Based on the evidential gaps described here, this study had three objectives. The first objective was to compare, within patient subgroups defined by key clinical characteristics, mean changes in NIS and NIS-LL total and subscale scores after 65 weeks between patients with ATTRv-PN receiving inotersen or placebo in the NEURO-TTR trial. The second objective was to estimate RD thresholds for NIS and NIS-LL total and subscale scores within this patient sample. The third objective was to examine the efficacy of inotersen for clinically meaningful slowing of neuropathic progression at the level of the individual patient by classifying responders using estimated RD thresholds, and then comparing the proportions of responders at week 65 between patients receiving inotersen and those receiving placebo.

## Methods

### Data source

Data for this study are from the NEURO-TTR trial, a phase 3, multinational, multicenter, randomized, placebo-controlled, double-blinded study of inotersen for the treatment of ATTRv-PN (ClinicalTrials.gov ID: NCT01737398) [8]. Adult patients with ATTRv-PN were randomized in a 2:1 ratio to receive 300 mg subcutaneous inotersen sodium or matching placebo once weekly for 65 weeks. A total of 172 patients (inotersen: 112; placebo: 60) were enrolled in the safety set, having received at least one dose of the study drug. Patients with amyloidosis confirmed by biopsy, a TTR variant confirmed by genotyping, and a NIS total score between 10 and 130 (inclusive) were eligible to participate; patients confined to wheelchairs or bedridden were not eligible to participate in the study.

### Ethical standards

The NEURO-TTR study protocol was approved by the relevant institutional review boards or local ethics committees and regulatory authorities. The study was conducted in accordance with Good Clinical Practice guidelines of the International Conference on Harmonization and the principles of the Declaration of Helsinki. All patients provided written informed consent to participate in the study.

### Target measures

#### Neuropathy Impairment Score (NIS)

The NIS is a clinician-rated measure of neuropathic progression that involves 37 bilateral assessments of the cranial nerves and limbs for muscle weakness, sensation loss, and decreased reflexes [13]. Assessments are conducted by a trained clinician who rates the degree of neuropathy at each site on scales ranging from 0 (normal nerve function) to 4 (paralysis) for cranial nerve and muscle weakness, and from 0 (normal) to 2 (absent) for reflex and sensation tests. Ratings are summed to calculate a composite total score that ranges from 0 to 244. Subscale scores for the NIS include cranial nerves (range: 0 to 40), muscle weakness (range: 0 to 152), sensation loss (range: 0 to 32), and decreased reflexes (range: 0 to 20). Higher NIS total and subscale scores reflect greater neuropathic impairment.

#### NIS-lower limbs (NIS-LL)

The NIS-LL is a subset of 14 NIS assessments specific to neuropathy in the lower limbs. A composite NIS-LL total score ranges from 0 to 88, with subscale scores for muscle weakness (range: 0 to 64), sensation loss (range: 0 to 16), and decreased reflexes (range: 0 to 8). Higher NIS-LL scores indicate greater neuropathic impairment of the lower limbs.

The NIS (and thus the NIS-LL item subset) was administered at baseline and week 65 visits. At each of these visits, the NIS was administered twice, with the two assessments recommended to occur on consecutive days. The two assessments at each visit were averaged. If only one assessment was conducted, then values from the single assessment were used. When possible, each patient was assessed by the same neurologist for all visits. Neurologists trained to administer the NIS/NIS-LL used standard procedures and equipment (e.g., cotton wool, pins, tuning fork, reflex hammer) and were instructed to consider abnormal nerve function in the context of the patient’s age, sex, weight, height, and overall physical fitness.

### Statistical analyses

This analysis was exploratory and conducted post hoc. The study population was the full analysis set (FAS), which included all randomized patients who had at least one dose of the study drug and at least one post-baseline efficacy assessment (*N* = 165). NIS and NIS-LL data from baseline and week 65 visits were used for this analysis. Due to very few patients in the study showing signs of cranial nerve impairment, this subscale of the NIS was not analyzed [9].

#### Evaluation of treatment benefit for Inotersen within patient subgroups

Treatment differences in least-squares (LS) mean change in NIS and NIS-LL scores from baseline to week 65 were examined for the FAS, as well as within patient subgroups defined by the following clinical characteristics: genetic mutation (V30M, non-V30M), familial amyloid polyneuropathy (FAP) disease stage (Stage 1 [ambulatory without assistance], Stage 2 [ambulatory with assistance of cane or walker]) [14], previous treatment status with tafamidis and/or diflunisal (pretreatment, no pretreatment), cardiomyopathy (CM) status (CM, no CM), and age of symptom onset (early [< 50 years], late [≥50 years]). Treatment differences in mean changes were analyzed using mixed-effects models for repeated measures (MMRM). Specifications of MMRM for analyses of the FAS included fixed categorical effects for treatment, time, randomization stratification factors (i.e., presence/absence of previous treatment with tafamidis and/or diflunisal; FAP Stage 1 or Stage 2; and V30M or non-V30M mutation), and treatment-by-time interaction, with fixed covariates for the baseline value and baseline-by-time interaction. Model specifications for analyses of subgroups included fixed categorical effects for treatment, time, randomization stratification factors, treatment-by-time interaction, treatment-by-subgroup interaction, and treatment-by-time-by-subgroup interaction, with fixed covariates for the baseline value and baseline-by-time interaction. Note that when subgroups included a stratification factor, that stratification factor was not included in the model. For example, when examining treatment differences within V30M and non-V30M subgroups, this factor was not included as a fixed categorical effect in the model.

The magnitude of treatment effects was also assessed using effect sizes for standardized mean differences, expressed as Cohen’s *d*, and interpreted according to Cohen’s published guidelines (*d* = 0.2, small effect; *d* = 0.5, medium effect; *d* = 0.8, large effect) [15].

#### Estimation of responder definition thresholds

Because ATTRv-PN is a progressive disease with a treatment goal of slowing or stabilizing neuropathy, rather than reversing it, as in previous studies estimating or applying RD thresholds for measures of neuropathic impairment in these patients [11, 16], responders in this study were defined as patients who did not exhibit clinically meaningful progression of neuropathic impairment, as measured by increases in NIS and NIS-LL scores after 65 weeks of treatment. RD thresholds for NIS-LL total and muscle weakness subscale scores were estimated using both anchor-based and distribution-based methods. RD thresholds for NIS muscle weakness and reflex subscales, as well as for the NIS-LL reflex subscale, were estimated using distribution-based methods only, as there were no appropriate anchor measures available for these outcomes. Table 1 provides a schematic for approaches and methods used to estimate RD thresholds for NIS and NIS-LL total and subscale scores.

**Table 1:** Summary of methods used to estimate responder definition (RD) thresholds

**Anchor-based Approaches**	**Target Measures**
➣ Mean Change *Mean change in NIS/NIS-LL scores for patients who did not exhibit meaningful worsening on the anchor* ➣ Linear Regression *Linear regression models with change in NIS/NIS-LL scores as the outcome and change in the anchor (LLF/PND) as the predictor* ➣ Receiver Operating Characteristic Curve *Receiver operating characteristic curves to identify the optimal cut-off point on NIS/NIS-LL scores for classifying patients showing meaningful worsening or not based on the anchor measure*	**Anchor:** *Polyneuropathy Disability (PND) score* • NIS Total • NIS Sensation • NIS-LL Sensation **Anchor:** *Lower Limb Function (LLF) test* • NIS-LL Total • NIS-LL Muscle Weakness
**Distribution-based Approaches**	**Target Measures**
➣ Effect Size *Group difference or change over time relative to the standard deviation at baseline* ➣ Standard Error of Measurement *Measurement error of a scale based on the standard deviation of baseline scores and the scale’s intra-rater reliability* ➣ Standardized Response Mean *Group difference or change over time relative to the standard deviation of change scores*	• All NIS and NIS-LL total and subscales

*Abbreviations*: *NIS* Neuropathy Impairment Score, *NIS-LL* Neuropathy Impairment Score – Lower Limbs

##### Anchor-based approaches

Anchor-based approaches estimate RD thresholds based on the correspondence between changes in the target measure and in an anchor measure. An anchor measure is an independent criterion measure for which there are clearly defined indicators for interpreting change in a patient’s clinical health. Appropriate anchor measures assess similar constructs as those captured by the target measure, and changes in the anchor should have at least a moderate statistical association with the target; a correlation ≥|0.30| between changes in the target measure and any anchor measure is recommended [17].

One measure was identified as an appropriate anchor for NIS total score and sensation subscales for both the NIS and NIS-LL: the Polyneuropathy Disability score (PND). The PND is a clinician-rated classification of patients into one of five stages of ambulatory disability: Stage I, indicating sensory disturbances in limbs without motor impairment; Stage II, indicating difficulty walking without the need of a walking aid; Stage IIIa, for which one stick or one crutch is required for walking; Stage IIIb, for which two sticks or two crutches are required for walking; and Stage IV, for patients who are confined to a wheelchair or bedridden [18]. An increase of one point on the PND can be interpreted as a clinically meaningful change. The PND was administered at baseline and week 65 visits. Spearman rank-order correlations between the changes from baseline to week 65 in PND score and changes in NIS total score and the NIS and NIS-LL sensation subscales were 0.30, 0.31, and 0.31, respectively, all of which were statistically significant (*p* < 0.001), supporting the use of the PND as an anchor measure for all three of these measures. Correlations between PND scores and all other NIS and NIS-LL measures were < 0.30.

A second measure was identified as an appropriate anchor for the NIS-LL total score and muscle weakness subscale: the lower limb function test (LLF). The LLF is a 3-item clinician assessment of a patient’s ability to walk on their toes, walk on their heels, and stand from a kneeling position [19]. Each item is assessed as normal (coded as 0) or abnormal (coded as 1), and are assessed bilaterally, yielding an LLF score ranging from 0 to 6, with higher scores indicating greater neuropathic impairment. An increase of two points on the LLF, representing bilateral change, can be interpreted as clinically meaningful. The LLF was administered at baseline and week 65 visits. Spearman rank-order correlations between the changes from baseline to week 65 in LLF score and changes in NIS-LL total and NIS-LL muscle weakness were 0.35 and 0.32, respectively, all of which were statistically significant (*p* < 0.001), supporting the use of the LLF as an anchor measure for both. Correlations between LLF scores and all other NIS and NIS-LL measures were < 0.30.

No appropriate anchor measure from the NEURO-TTR trial was identified for the NIS muscles weakness subscale or for NIS or NIS-LL reflex subscales, as no other clinician-rated assessment of neuropathic impairment that was conceptually related to these outcomes had straightforward interpretation of what would indicate clinically meaningful improvement.

Three anchor-based methods were used to estimate RD thresholds for the NIS/NIS-LL measures from corresponding anchors. First, the mean change in NIS/NIS-LL scores for patients who did not exhibit meaningful worsening on the anchor (i.e., < 2-point increase on the LLF, or < 1-point increase on the PND) was subtracted from mean change in these scores for patients with a ≥ 2-point increase on the LLF/≥1-point increase on the PND [17, 20, 21]. Second, linear regression models were conducted, with change in NIS/NIS-LL scores as the outcome and change in LLF/PND as the predictor [22]. The β-coefficient from each model represents the change in NIS/NIS-LL score corresponding to a 2-point increase in LLF/1-point increase in PND. Third, receiver operating characteristic (ROC) curves were used to identify the optimal cut-off point on NIS/NIS-LL scores for classifying patients showing meaningful worsening or not based on the anchor measure (i.e., ≥2-point increase vs. < 2 increase on LLF/≥1-point increase vs. < 1 increase on PND) [20, 23–25]. The optimal cut-off point was defined using the Index of Union method, which identifies the point at which the sensitivity and specificity values are simultaneously closest to the value of the area under the curve [26].

##### Distribution-based approaches

Distribution-based approaches estimate RD thresholds based on statistics that describe the variation and precision of scores, such as a scale’s standard deviation (SD) and reliability, to assess the amount of difference or change on a measure that cannot be explained by measurement error and is considered to reflect a clinically meaningful treatment effect. Three distribution-based statistics were used in the estimation of RD thresholds on all NIS and NIS-LL scores: effect size (ES), standardized response mean (SRM), and standard error of measurement (SEM). The mean of these estimates was then used as the recommended RD threshold.

The ES has long been used to interpret the magnitude of difference between groups or change over time in education, psychology, and health outcomes research [15, 27]. Group difference or change over time is measured against the standard deviation at baseline (SD_Baseline_). For this analysis, the ES was set to 0.5, which is considered to indicate a medium-sized effect and has been shown to closely align with estimates of RD thresholds for other clinical outcome assessments (COAs) used across many health conditions [28, 29]. This value is then multiplied by the SD_Baseline_.

The SRM is another statistic used to interpret group differences and change over time, this time measured against the standard deviation of change from baseline (SD_Change_). The SRM was set to 0.5 for this analysis, which was then multiplied by the SD_Change_.

The SEM captures measurement error of a scale based upon variability of scores (SD_Baseline_) and the scale’s reliability. Researchers have observed that the SEM of a measure had a magnitude similar to RD thresholds estimated using anchor-based approaches [30, 31]. Intra-rater reliability was used in this analysis, assessed across the two administrations of the NIS/NIS-LL conducted by the same rater (recommended to occur on consecutive days) during the baseline visit, calculated using intraclass correlation coefficient (ICC). ICC was calculated using Shrout and Fleiss’ [1, 2] model [32], a two-way random effects model appropriate for capturing intra-rater reliability when a single rater performs two assessments of the same target, when assuming that scores from raters are generalizable to the population of raters [33]. The SEM is then calculated by multiplying SD_Baseline_ by the square root of one minus the ICC.

##### Recommended RD threshold based on triangulation

Triangulation across multiple estimates, which is generally considered best practice [17, 34–36], was used to establish a recommended RD threshold for each scale. The recommended RD threshold was calculated as the mean across all estimates.

#### Responder analysis

Responder analysis was conducted to evaluate the efficacy of inotersen for slowing neuropathic progression at the patient level. For each NIS and NIS-LL measure, the proportion of patients classified as responders (i.e., patients whose score increased by less than the recommended RD threshold after 65 weeks of treatment) were compared between treatment groups using odds ratios (OR) with 95% confidence intervals (CI), and Fisher’s exact tests (two-tailed α) for statistical significance. Because these analyses were exploratory, no adjustments were made to the familywise Type 1 error rate for multiple comparisons.

To be consistent with the efficacy analysis of NIS/NIS-LL in the NEURO-TTR study, no imputation of missing values was performed in the primary responder analysis. As such, the responder analysis used a complete-case analysis, in which only patients with non-missing scores at the week 65 visit were included.

#### Empirical cumulative distribution function (eCDF) curves

Empirical cumulative distribution function (eCDF) curves were plotted to visually represent the percentages of patients with changes in NIS or NIS-LL total score below each observed change score from baseline to week 65. Treatment differences in the percentage of patients at each threshold of change were explored by plotting separate curves for treatment and placebo groups and examining the distance between the curves on the y-axis at each point of the x-axis.

## Results

### Patient characteristics

Baseline patient characteristics and NIS/NIS-LL total and subscale scores by treatment arm are reported in Table 2. Patients in each treatment group were very similar in age and sex distribution, as well as on other key clinical characteristics such as mutation type, FAP stage, previous treatment status, cardiomyopathy, and age of symptom onset. Differences between treatment groups were not statistically significant on any patient characteristics or NIS/NIS-LL scores.

**Table 2:** Baseline patient characteristics in the NEURO-TTR trial, full analysis set (*N* = 165)

	Inotersen (*n* = 106)	Placebo (*n* = 59)
Age, mean (SD)	59.6 (12.4)	59.4 (14.1)
Sex, N (%)		
Male	75 (71)	41 (70)
Female	31 (29)	18 (30)
Mutation Type, N (%)		
V30M	54 (51)	33 (56)
Non-V30M	52 (49)	26 (44)
FAP stage, N (%)		
Stage 1 (ambulatory without assistance)	71 (67)	42 (71)
Stage 2 (ambulatory with assistance)	35 (33)	17 (29)
Previous treatment status^a^, N (%)		
Pretreatment	62 (59)	35 (59)
No pretreatment	44 (41)	24 (41)
Cardiomyopathy status, N (%)		
Cardiomyopathy	70 (66)	32 (54)
No cardiomyopathy	36 (34)	27 (46)
Age of symptom onset, N (%)		
Early onset	31 (29)	20 (34)
Late onset	75 (71)	39 (66)
NIS, mean (SD)		
Total	46.6 (25.7)	43.4 (24.7)
Muscle Weakness	21.2 (17.5)	20.0 (16.1)
Sensation	14.4 (6.3)	13.3 (6.9)
Reflexes	10.9 (6.0)	10.1 (6.4)
NIS-LL, mean (SD)		
Total	30.1 (15.5)	28.7 (16.0)
Muscle Weakness	13.9 (11.3)	13.4 (11.0)
Sensation	10.2 (4.0)	9.8 (4.5)
Reflexes	6.0 (2.3)	5.6 (2.7)

*Abbreviations*: *FAP* familial amyloid polyneuropathy, *NIS* Neuropathy Impairment Score, *NIS-LL* Neuropathy Impairment Score – Lower Limb, *SD* standard deviation

^a^Previous treatment with tafamidis and/or diflunisal

### Treatment differences in mean change for NIS and NIS-LL scores for clinical subgroups

**Fig. 1:**
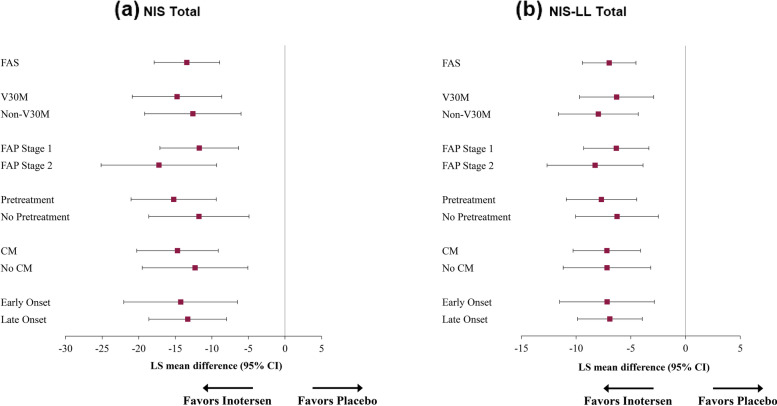
Treatment Differences in NIS (**a**) and NIS-LL (**b**) Total Mean Change Scores from Baseline to Week 65. Note: Means in purple ink are statistically significantly different from 0 (*p* < 0.05). Abbreviations: CI, confidence interval; CM, cardiomyopathy; FAP, familial amyloid polyneuropathy; FAS, full analysis set; LS, least-squares; NIS, Neuropathy Impairment Score; NIS-LL, Neuropathy Impairment Score – Lower Limb

**Fig. 2:**
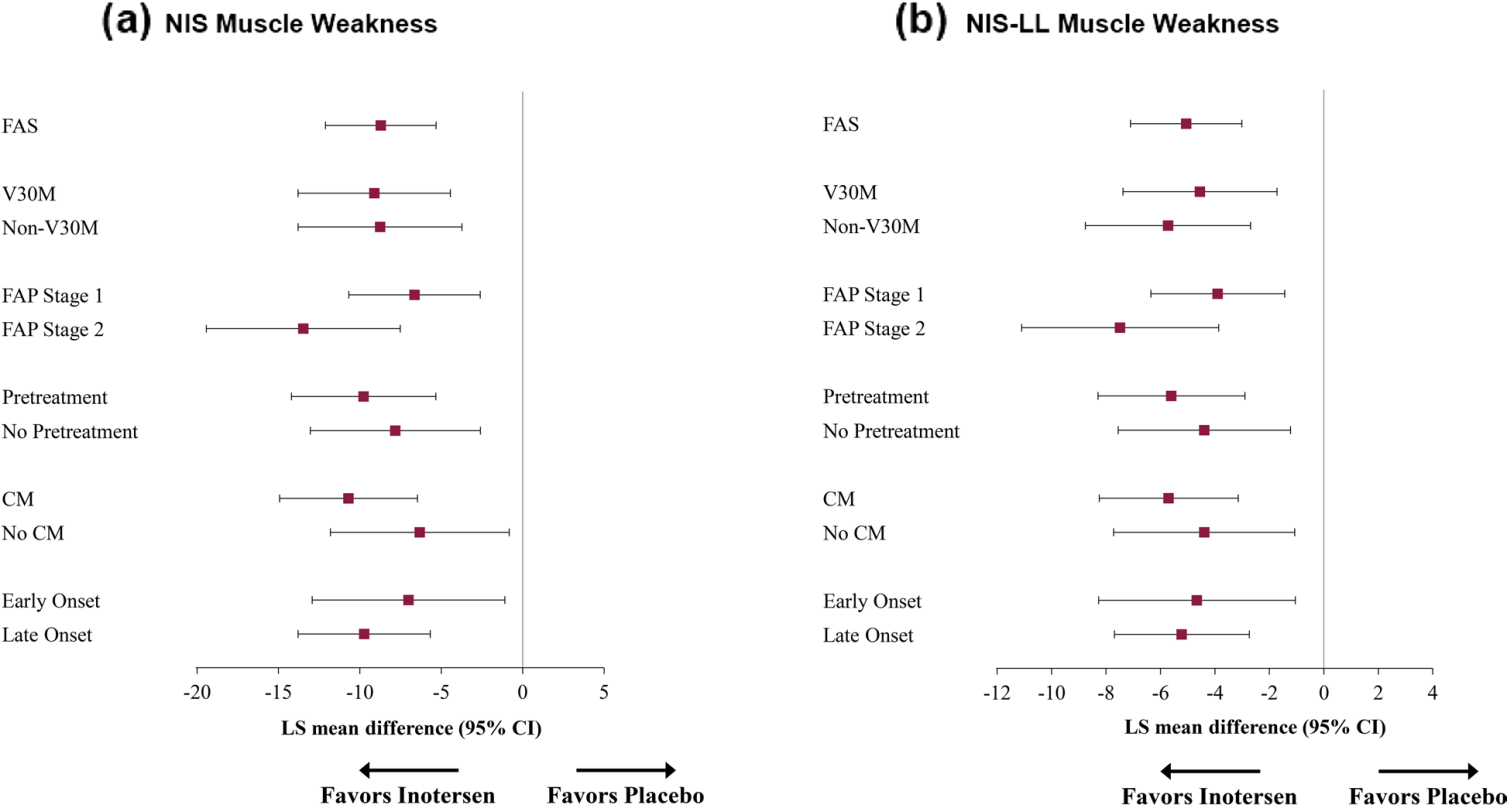
Treatment Differences in NIS (**a**) and NIS-LL (**b**) Muscle Weakness Domain Change Scores from Baseline to Week 65. Note: Means in purple ink are statistically significantly different from 0 (*p* < 0.05). Abbreviations: CI, confidence interval; CM, cardiomyopathy; FAP, familial amyloid polyneuropathy; FAS, full analysis set; LS, least-squares; NIS, Neuropathy Impairment Score; NIS-LL, Neuropathy Impairment Score – Lower Limb

**Fig. 3:**
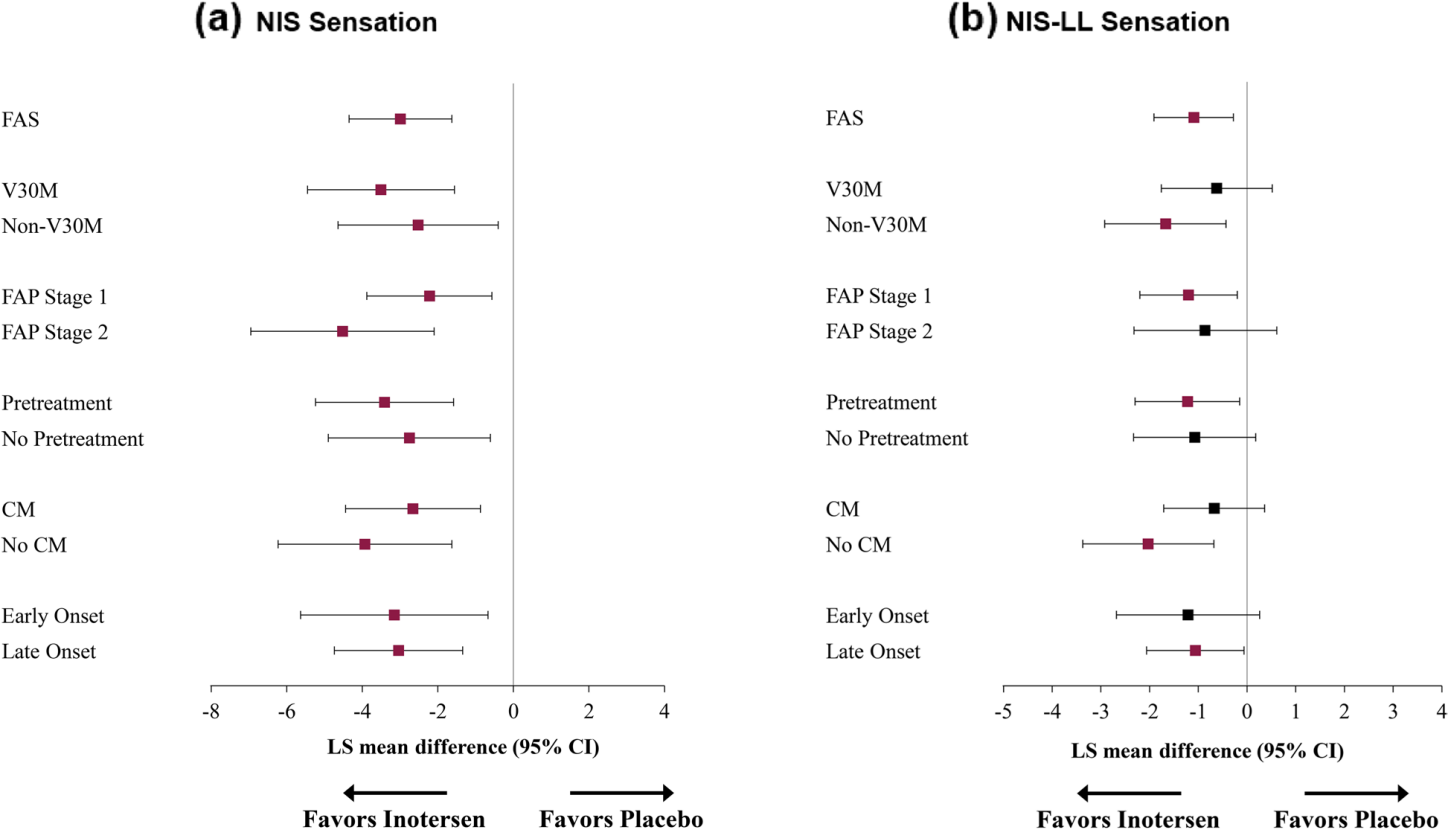
Treatment Differences in NIS (**a**) and NIS-LL (**b**) Sensation Domain Change Scores from Baseline to Week 65. Note: Means in purple ink are statistically significantly different from 0 (*p* < 0.05). Abbreviations: CI, confidence interval; CM, cardiomyopathy; FAP, familial amyloid polyneuropathy; FAS, full analysis set; LS, least-squares; NIS, Neuropathy Impairment Score; NIS-LL, Neuropathy Impairment Score – Lower Limb

**Fig. 4:**
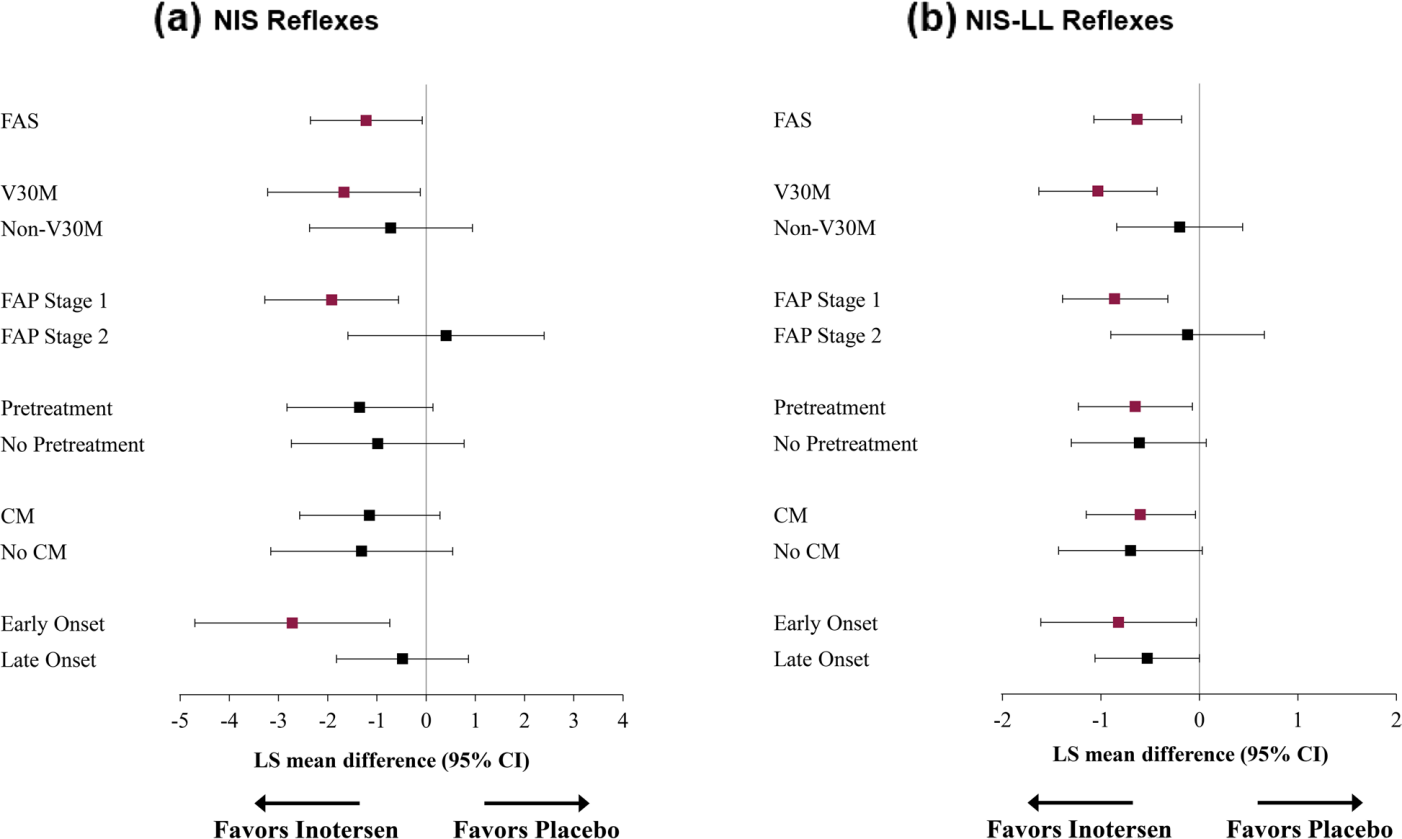
Treatment Differences in NIS (**a**) and NIS-LL (**b**) Reflexes Domain Change Scores from Baseline to Week 65. Note: Means in purple ink are statistically significantly different from 0 (*p* < 0.05). Abbreviations: CI, confidence interval; CM, cardiomyopathy; FAP, familial amyloid polyneuropathy; FAS, full analysis set; LS, least-squares; NIS, Neuropathy Impairment Score; NIS-LL, Neuropathy Impairment Score – Lower Limb

Treatment differences in LS mean changes on NIS and NIS-LL total and subscale scores from baseline to 65 weeks for key clinical subgroups are presented in Figs. 1, 2, 3 and 4.

Statistically significant mean differences for NIS total scores (Fig. 1a) ranged from − 11.7 (FAP Stage 1) to − 17.2 points (FAP Stage 2), with large treatment effects within all subgroups (*d*s ranged from − 0.89 to − 1.30). Similar findings were observed for the NIS-LL total score (Fig. 1b), with statistically significant mean differences ranging from − 6.3 (V30M, FAP Stage 1, and No Pretreatment) to − 8.3 (FAP Stage 2) and large treatment effects (*d*s ranged from − 0.87 to − 1.12).

For the NIS muscle weakness subscale (Fig. 2a), statistically significant mean differences ranged from − 6.3 (No CM) to − 13.5 points (FAP Stage 2), with medium-to-large effects (*d*s ranged from − 0.66 to − 1.35). Statistically significant mean differences on the NIS-LL muscle weakness subscale (Fig. 2b) ranged from − 3.9 (FAP Stage 1) to − 7.5 (FAP Stage 2), also with medium-to-large treatment effects (*d*s ranged from − 0.65 to − 1.23).

Overall, LS mean change from baseline on the NIS sensation subscale (Fig. 3a) after 65 weeks of treatment followed the same pattern as the NIS total score and muscle weakness subscale. Statistically significant differences in mean change on NIS sensation subscale scores ranged from − 2.2 (FAP Stage 1) to − 4.5 points (FAP Stage 2), with medium-to large treatment effects (*d*s ranged from − 0.55 to − 1.11). Treatment differences in LS mean change for the NIS-LL sensation subscale (Fig. 3b) were statistically significant for Non-V30M, FAP Stage 1, Pretreatment, No CM, and Late Onset subgroups, ranging from − 1.1 (Late Onset) to − 2.0 points (No CM), with small-to-large treatment effects (*d*s ranged from 0.43 to − 0.87).

Statistically significant differences in mean change on the NIS reflexes subscale (Fig. 4a) were observed for V30M, FAP Stage 1, and Early Onset subgroups, ranging from − 1.7 (V30M) to − 2.7 points (Early Onset) and with medium-to-large treatment effects (*d*s ranged from − 0.51 to − 0.84). For the NIS-LL reflexes subscale (Fig. 4b), statistically significant differences in mean change were observed for the V30M, FAP Stage 1, Pretreatment, CM, and Early Onset subgroups, ranging from − 0.6 (CM) to − 1.0 points (V30M), with small-to-large effects (*d*s ranged from − 0.44 to − 0.82).

### Responder definition estimates for NIS and NIS-LL scores

Scale properties (SD_baseline_, SD_change_, and ICCs for reliability) and RD threshold estimates for NIS and NIS-LL total and subscales, including the mean of the estimates (i.e., the recommended RD threshold) are presented in Table 3. ICCs for intra-rater reliability at baseline ranged from 0.88 (NIS-LL reflexes) to 0.99 (NIS total and muscle weakness). Means of RD thresholds estimated using anchor-based and distribution-based methods were similar for each measure for which both were estimated: 8.6 vs. 7.7, respectively, for NIS total; 2.4 vs 2.5 for NIS sensation; 4.8 vs. 4.6 for NIS-LL total; 3.5 vs. 3.4 for NIS-LL muscle weakness; and 1.1 vs. 1.5 for NIS-LL sensation. Among anchor-based methods, RD threshold estimates were larger for mean change methods than for linear regression and ROC curve methods. For distribution-based methods, RD threshold estimates based on ES were consistently the largest, followed by estimates based on SRM, and generally smallest for SEM. Relative magnitude of variation among RD threshold estimates was particularly evident for the NIS muscle weakness subscale, with a range of 6.6 points and a relatively large coefficient of variation (CV) of 63%, and for NIS-LL total and muscle weakness scores, with ranges of 5.8 and 4.2 and CVs of 52 and 53%, respectively. CVs were smallest for NIS sensation (29%) and NIS-LL reflex subscales (25%).

**Table 3:** Estimates of responder definition thresholds for NIS and NIS-LL total and subscale scores

Instrument/Score	Scale Properties			Anchor-based estimates			Distribution-based estimates			Recommended RD threshold^**a**^
	Reliability	SD_**baseline**_	SD_**change**_	Mean change	Linear regression	ROC	ES	SEM	SRM	
*NIS*										
Total	0.99	25.3	14.6	11.8^b^	7.0^b^	6.9^b^	12.7	3.0	7.3	8.1
Muscle weakness	0.99	17.0	10.7	–	–	–	8.5	1.9	5.4	5.3
Sensation	0.92	6.5	4.3	3.3^b^	2.0^b^	1.8^b^	3.3	1.9	2.2	2.4
Reflex	0.95	6.1	3.5	–	–	–	3.1	1.3	1.7	2.0
*NIS-LL*										
Total	0.98	15.7	7.8	7.0^c^	2.0^c^	5.3^c^	7.8	2.2	3.9	4.7
Muscle weakness	0.98	11.2	6.3	5.2^c^	1.4^c^	3.8^c^	5.6	1.4	3.1	3.4
Sensation	0.91	4.2	2.5	1.8^b^	1.2^b^	0.3^b^	2.1	1.3	1.2	1.3
Reflex	0.88	2.4	1.6	–	–	–	1.2	0.8	0.8	0.9

*Abbreviations*: *ES* effect size, *LLF* Lower Limb Function Test, *NIS* Neuropathy Impairment Score, *NIS-LL* Neuropathy Impairment Score – Lower Limb, *PND* Polyneuropathy Disability score, *RD* responder definition, *ROC* receiver operating characteristic, *SD* standard deviation, *SEM* standard error of measurement, *SRM* standardized response mean

^a^Recommended RD threshold was calculated as the mean of RD estimates

^b^RD threshold estimate was calculated using PND as the anchor measure

^c^RD threshold estimate was calculated using LLF as the anchor measure

### Responder analysis for NIS and NIS-LL scores

**Fig. 5:**
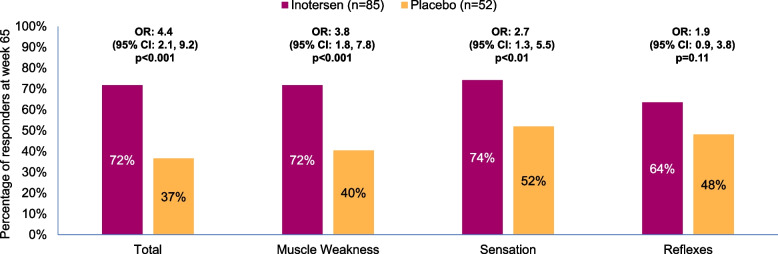
Proportion of Responders at Week 65 by Treatment Arm for NIS Total and Subscale Scores. Abbreviations: CI, confidence interval; NIS, Neuropathy Impairment Score; OR, odds ratio

**Fig. 6:**
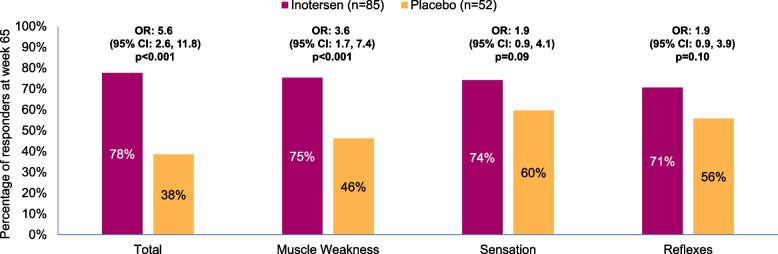
Proportion of Responders at Week 65 by Treatment Arm for NIS-LL Total and Subscale Scores. Abbreviations: CI, confidence interval; NIS-LL, Neuropathy Impairment Score – Lower Limb; OR, odds ratio

Results from the responder analysis for all NIS and NIS-LL scores are shown in Figs. 5 and 6, respectively. For each NIS score, the proportion of responders (i.e., patients whose scores increased by less than the RD threshold) at week 65 was larger among patients treated with inotersen than patients who received placebo. Among patients treated with inotersen, the proportion of responders on NIS total and subscale scores ranged from 64 to 74%, compared to 37 to 52% among patients who received placebo, with statistically significant treatment differences for total (OR = 4.4), muscle weakness (3.8), and sensation (2.7), all *p* < 0.01, but not for reflexes (1.9, *p* = 0.11).

For each NIS-LL score, the proportion of responders at week 65 was larger among patients receiving inotersen (range: 71–78%) than patients receiving placebo (38–60%). Statistically significant treatment differences in proportion of responders were observed for NIS-LL total (OR = 5.6) and muscle weakness (3.6), *p*<0.001 for both, but not for sensation (1.9, *p* = 0.09) or reflexes (1.9, *p* = 0.10).

### eCDF curves

The eCDF curves for changes in NIS and NIS-LL total scores from baseline to week 65 within each treatment arm are presented in Supplementary Figs. 1 and 2, respectively. A description of these results, which supports treatment benefit on both measures across the entire range of change, is reported in the Supplementary Appendix.

## Discussion

Treatment differences on mean change and response on the NIS and NIS-LL demonstrate the efficacy of inotersen for slowing neuropathic progression in patients with ATTRv-PN. Treatment benefits of inotersen were observed for NIS and NIS-LL total and muscle weakness scores, as well as NIS sensation scores, across patient subgroups based on genetic mutation, FAP disease stage, previous treatment, cardiomyopathy status, and age of symptom onset. Responder analysis demonstrated that treatment benefits on the NIS and NIS-LL were experienced by a majority of patients with ATTRv-PN treated with inotersen (64–78%), which would indicate that treatment differences in mean changes at the group level were not due to outliers in either treatment arm. Patients treated with inotersen were significantly less likely to experience clinically meaningful progression of neuropathic symptoms, including muscle weakness and sensation, than patients who received placebo. For example, 72 and 78% of patients treated with inotersen did not experience clinically meaningful worsening on NIS and NIS-LL total scores, respectively, compared to 37 and 38% of patients who received placebo. Visual inspection of eCDF curves for change in NIS and NIS-LL total scores after 65 weeks reinforced these findings, revealing treatment differences favoring inotersen across the range of observed change scores on both measures.

Treatment benefits of inotersen on the NIS-LL sensation subscale, and for both NIS and NIS-LL reflexes subscales, were observed in some, but not all, patient subgroups. For some of these subgroups where treatment differences were not statistically significant, the magnitudes of mean differences and ESs were similar to those observed in the FAS. Thus, in some of these cases, the lack of significant treatment differences could have been at least partially an effect of insufficient statistical power to detect a true treatment effect due to small sample sizes in some subgroups. Additionally, while patients treated with inotersen showed little-to-no progression on these subscales, patients who received placebo also experienced less progression on these subscales relative to the others, as shown from analyses at both the group and patient level.

Patients with ATTRv-PN in FAP Stage 2 experienced the largest benefit from treatment with inotersen on the NIS and NIS-LL total scores and muscle weakness subscales. As would be expected, all NIS/NIS-LL total and domain scores are substantially worse at baseline for patients in FAP Stage 2 than for those in FAP Stage 1. Patients in FAP Stage 2 also showed the most progression in most of these measures, as evidenced by the larger increases in score compared to patients in FAP Stage 1 for the placebo arm on NIS total score (mean increase of 24.1 points for FAP Stage 2 vs. 15.5 points for FAP Stage 1, *p* = 0.031, *d* = 0.67), muscle weakness (18.2 vs. 9.6, *p* = 0.005, *d* = 0.88), and sensation (4.8 vs. 2.0, *p* = 0.017, *d* = 0.74), as well as for NIS-LL total score (11.6 vs. 8.6, *p* = 0.152, *d* = 0.44) and muscle weakness (10.2 vs. 5.9, *p* = 0.014, *d* = 0.76). At the same time, patients in FAP Stage 2 showed the smallest treatment benefit on the NIS and NIS-LL reflexes domain. Again, patients with FAP Stage 2 disease had substantial reflex deficits at baseline compared to patients with FAP Stage 1 disease: for those in the placebo arm, baseline mean scores were 15.2 vs 8.0 on NIS reflexes, and 7.7 vs. 4.4 on NIS-LL reflexes, both *p* < 0.001, both *d* > 1.2. However, unlike for other domains, only minimal further progression was observed at 65 weeks for patients in this subgroup receiving placebo, with a mean increase of only 1.1 points on the NIS reflexes domain (compared to 3.3 points for FAP Stage 1 patients, *p* = 0.040, *d* = 0.63) and 0.3 points on the NIS-LL reflexes domain (compared to 1.3 points for FAP Stage 1 patients, *p* = 0.013, *d* = 0.78) These data may indicate that patients with FAP Stage 2 disease had experienced full loss of reflexes prior to treatment, and as such received no treatment benefit. The case for earlier treatment of these patients then is quite clear.

When interpreting treatment differences across subgroups, it is important to take into account the fact that these groups are not independent, but rather some of these patient characteristics covary, leading to substantial overlap in patients across some subgroups. A post hoc analysis, using χ^2^ tests of independence, found statistically significant associations among several patient subgroups, particularly among those based on genetic mutation, FAP stage, cardiomyopathy status, and age of symptom onset. For example, 75% of patients with V30M had early onset of symptoms, while the majority of patients with non-V30M (57%) had late onset of symptoms (perhaps surprisingly, there was no statistical association between patients’ CM status and whether they had received pretreatment with tafamidis and/or diflunisal). These overlaps among patient subgroups may account for similarities in treatment effects, such as finding significant treatment benefit on reflexes for both V30M and early onset patients, but not for non-V30M and late onset patients.

The ability to evaluate the clinical significance of treatment differences on COA measures such as the NIS/NIS-LL is limited without the availability of RD thresholds. This study contributes interpretation guidelines for change in NIS and NIS-LL scores by providing empirically based estimates of RD thresholds that represent clinically meaningful progression of neuropathic symptoms. The RD thresholds recommended here are derived from multiple estimates, including three distribution-based estimates for all scores, and three anchor-based estimates for NIS total and sensation scores, and NIS-LL total, muscle weakness, and sensation subscale scores. The similarities in magnitudes of estimated values across the distribution-based and anchor-based estimates for scales in which both methods were used would seem to support that the distribution-based estimates for the remaining scales are similar to estimates that would be derived if appropriate anchors for those scales were available.

The recommended RD thresholds for the NIS and NIS-LL total scores (8.1 and 4.7 points, respectively), are much larger than the 2-point RD threshold considered in some previous studies [11–13] and represent a greater change relative to the range of these scales (3.3% versus 0.8% on the NIS, and 5.3% versus 2.3% on the NIS-LL). The establishment of thresholds indicating meaningful change at the level of the individual patient could be used in clinical practice, as it would enable clinicians to track progression of neuropathic impairments more accurately among their patients with ATTRv-PN, including evaluation of the effectiveness of treatments for stabilization. The establishment of these thresholds will also aid in the design and interpretation of data from clinical trials of patients with ATTRv-PN; these values could be used to determine the minimum sample size for a trial to be adequately powered to detect treatment differences in trials for which the NIS or NIS-LL is a key endpoint and would allow for conducting responder analyses that would inform evaluation of treatment benefit at the patient level.

This study had some limitations that should be noted. Due to the lack of suitable anchor measures for the NIS muscle weakness scores and for NIS and NIS-LL reflexes subscale scores, RD threshold estimates for these scales were derived entirely from distribution-based methods, which are typically considered to be inferior or secondary to anchor-based methods for estimating clinically meaningful change [17, 37–39]. Another limitation concerns the amount of variability across distribution-based estimates for RD thresholds. Distribution-based estimates for RD thresholds for NIS/NIS-LL total and muscle weakness subscale scores varied considerably, with estimates based on the ES up to four times larger than estimates based on the SEM.

An additional limitation of this study is that the threshold representing a meaningful change on a measure may vary as a function of a patient’s baseline severity, particularly for scales that are non-linear like those examined here [21, 40]. In the current sample, two-thirds of patients at baseline were able to walk unassisted (FAP Stage 1), and the remainder required the assistance of walkers or canes (FAP Stage 2). Patients at earlier and less severe stages of the disease have the potential to still experience more significant decline than patients whose disease has already progressed to later stages. The experience of decline itself may also carry different meaning for patients at earlier or later stages of disease progression. Due to this, these estimates for RD thresholds cannot be assumed to represent clinically important change for patients at all levels of disease severity and progression.

## Conclusion

In conclusion, this is the first research to examine the efficacy of inotersen on NIS and NIS-LL scores among subgroups of patients with ATTRv-PN based on key clinical characteristics, such as genetic mutation, FAP stage, pretreatment status, presence of cardiomyopathy, and age of onset. Additionally, RD thresholds for NIS and NIS-LL scores in patients with ATTRv-PN were estimated using anchor-based and/or distribution-based methods, allowing for improved evaluation of treatment benefit for slowing neuropathic impairment. The mean of these estimates was given as the recommended RD threshold for the total and subscale scores of the NIS (total: 8.1 points; muscle weakness: 5.3; sensation: 2.4; and reflexes: 2.0) and NIS-LL (total: 4.7 points; muscle weakness: 3.4; sensation: 1.3; and reflexes: 0.9). Patient-level responder analyses using these RD thresholds showed that inotersen provides a clinically meaningful benefit for limiting the progression of neuropathic impairment in patients with ATTRv-PN, particularly on measures of muscle weakness and sensation loss. These results support previous evidence demonstrating the efficacy of inotersen in this patient population.

## Supplementary Information

**Additional file 1.**

**Additional file 2: Sup Fig. 1.** Empirical distribution function curve for change in NIS total score from baseline to week 65 by treatment arm. Abbreviation: NIS, neuropathy impairment score.

**Additional file 3: Sup Fig. 2.** Empirical distribution function curve for change in NIS total score from baseline to week 65 by treatment arm. Abbreviation: NIS-LL, neuropathy impairment score – lower limb.

## Data Availability

The datasets used and/or analyzed during the current study are available from the corresponding author on reasonable request.

## Abbreviations

ATTRv: Hereditary transthyretin amyloidosis
ATTRv-PN: Hereditary transthyretin amyloidosis with polyneuropathy
CI: Confidence intervals
CM: Cardiomyopathy
COA: Clinical outcome assessment
CV: Coefficient of variation
eCDF: Empirical cumulative distribution function
ES: Effect size
FAP: Familial amyloid polyneuropathy
FAS: Full analysis set
ICC: Intraclass correlation coefficient
LLF: Lower limb function test
LS: Least-squares
MCID: Minimal clinically important difference
MMRM: Mixed-effects models for repeated measures
mNIS+ 7: Modified Neuropathy Impairment Score plus 7 nerve tests
NIS: Neuropathy Impairment Score
NIS-LL: Neuropathy Impairment Score – Lower Limbs
NIS + 7: Neuropathy Impairment Score plus 7 nerve tests
OR: Odds ratio
PN: Polyneuropathy
RD: Responder definition
ROC: Receiver operating characteristic
SD: Standard deviation
SEM: Standard error of measurement
SRM: Standardized response mean
TTR: Transthyretin
