# Supplementary material for: The impact of inotersen on Neuropathy Impairment Score in patients with hereditary transthyretin amyloidosis with polyneuropathy
Source: BMC Neurol. 2023 Mar 17;23:108. doi: 10.1186/s12883-023-03116-7 (PMC10022100; doi:10.1186/s12883-023-03116-7)
Supplement: Supplementary file 1 — Additional file 1. [file 12883_2023_3116_MOESM1_ESM.docx]

**eCDF curves**

The eCDF curves for changes in NIS total scores from baseline to week 65 within each treatment arm are presented in Supplementary Figure 1. Separation between the curves across the entire range of change indicates that larger percentages of patients treated with inotersen had change scores below each value than patients who received placebo. Treatment differences were largest between 5 and 25 points of change, where the percentages of patients treated with inotersen with change in scores below those points were approximately 30–40 percentage-points higher than for patients who received placebo.

[Supplementary Figure 1]

The eCDF curves for changes in NIS-LL total score from baseline to week 65 within each treatment arm are presented in Supplementary Figure 2. Apart from at the margins of the range of change scores, larger percentages of patients treated with inotersen can be observed with change scores below each threshold than patients who received placebo. Treatment differences were largest between 2 and 14 points of change, where the percentages of patients treated with inotersen with change in scores below those points were roughly 30 percentage-points higher than for patients who received placebo.

[Supplementary Figure 2]

**Sup Fig. 1** Empirical distribution function curve for change in NIS total score from baseline to week 65 by treatment arm

Abbreviation: NIS, Neuropathy Impairment Score

**Sup Fig. 2** Empirical distribution function curve for change in NIS-LL total score from baseline to week 65 by treatment arm

Abbreviation: NIS-LL, Neuropathy Impairment Score – Lower Limb
