# Supplementary figures and images for: The impact of inotersen on Neuropathy Impairment Score in patients with hereditary transthyretin amyloidosis with polyneuropathy
Source: BMC Neurol. 2023 Mar 17;23:108. doi: 10.1186/s12883-023-03116-7 (PMC10022100; doi:10.1186/s12883-023-03116-7)

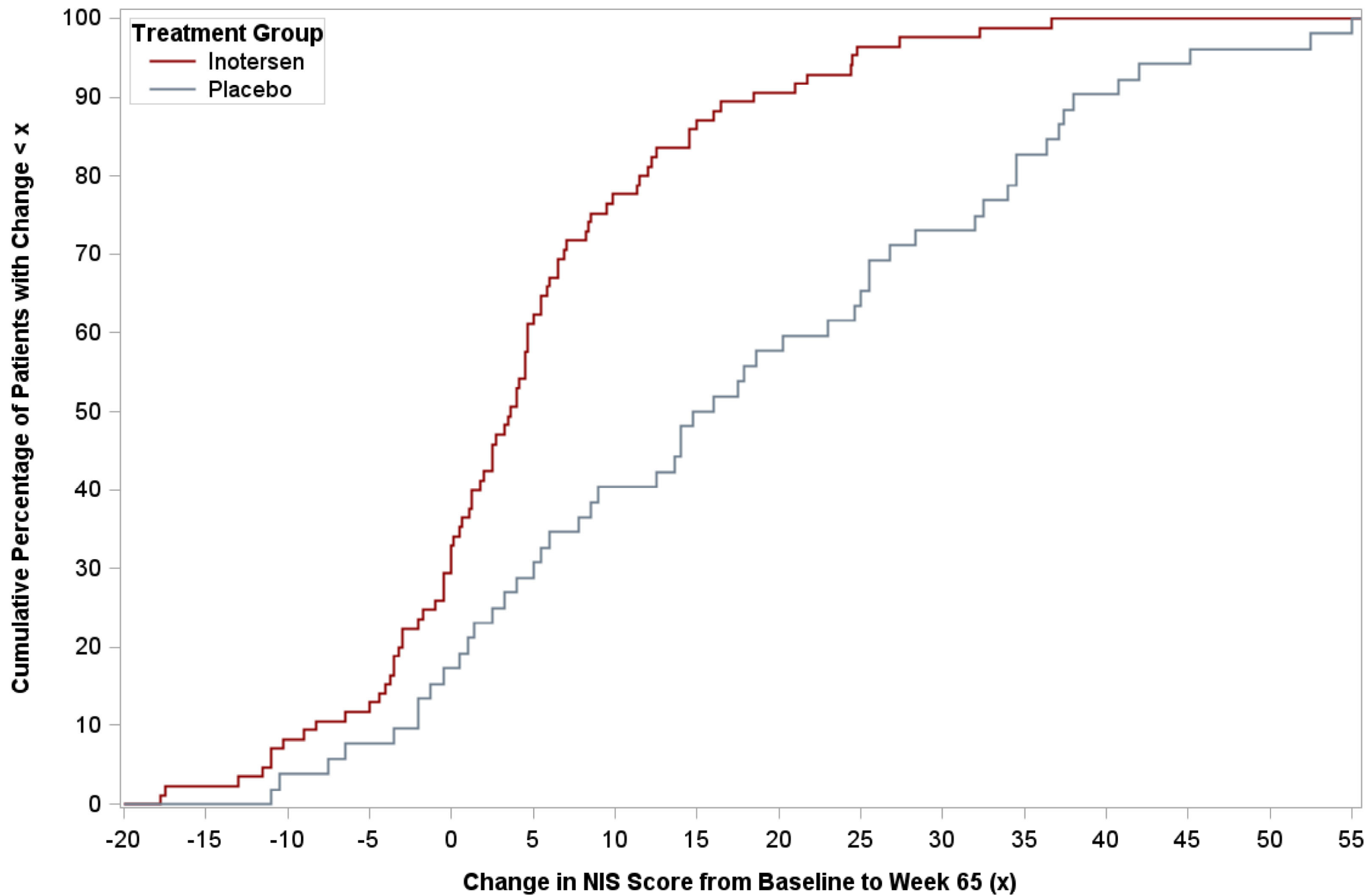

Supplement: Supplementary file 2 — Additional file 2: Sup Fig. 1. Empirical distribution function curve for change in NIS total score from baseline to week 65 by treatment arm. Abbreviation: NIS, neuropathy impairment score. [file 12883_2023_3116_MOESM2_ESM.pdf]

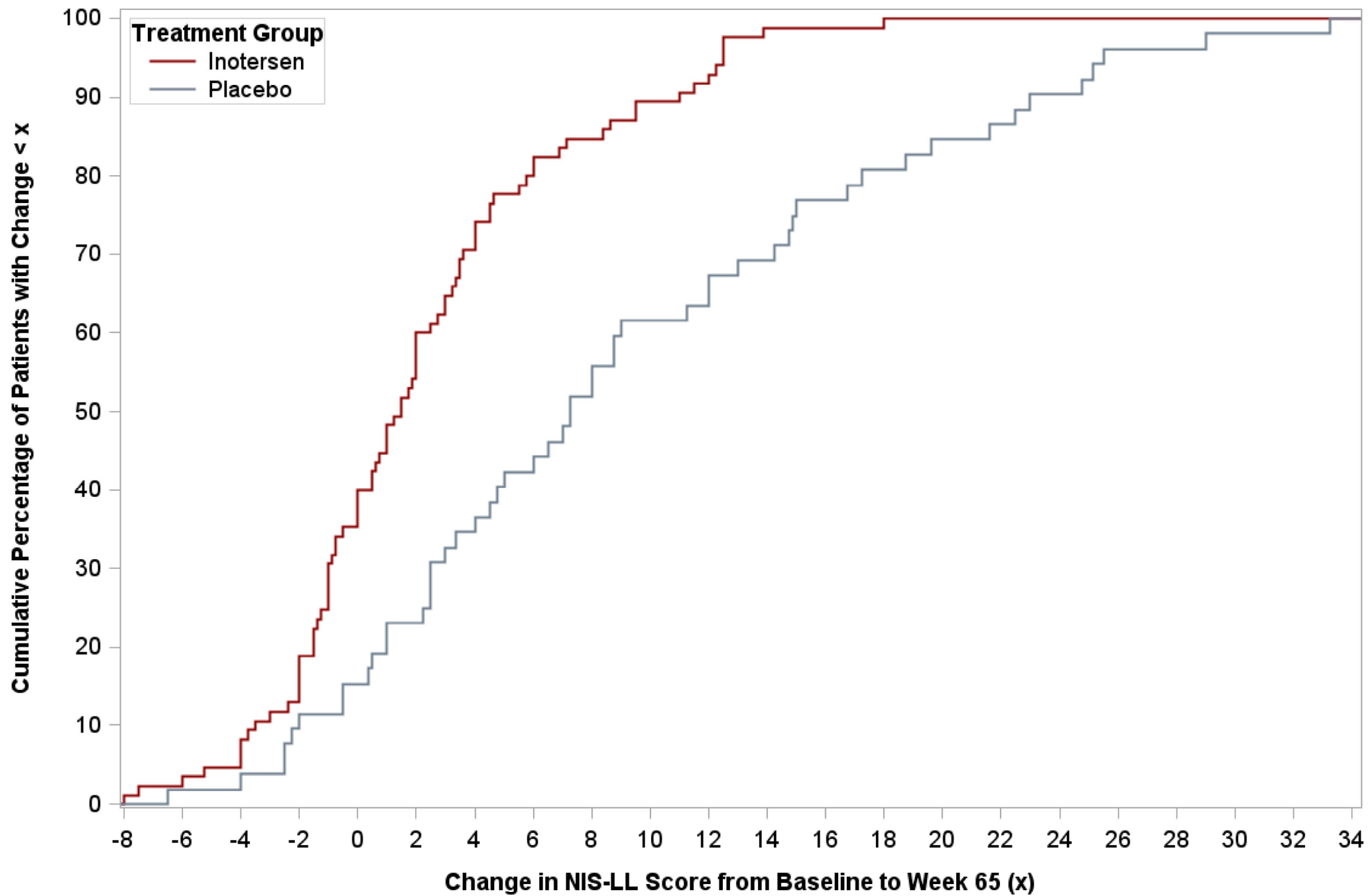

Supplement: Supplementary file 3 — Additional file 3: Sup Fig. 2. Empirical distribution function curve for change in NIS total score from baseline to week 65 by treatment arm. Abbreviation: NIS-LL, neuropathy impairment score – lower limb. [file 12883_2023_3116_MOESM3_ESM.pdf]
